# Pulmonary *Verruconis* Infection in an Immunocompetent Patient: A Case Report and Literature Review

**DOI:** 10.3390/jof11090634

**Published:** 2025-08-29

**Authors:** Lulu Xu, Lili Tao

**Affiliations:** 1 Department of Pathology, Microbiology, and Immunology, Vanderbilt University Medical Center, Nashville, TN 37228, USA; 2 Chongqing Clinical Research Center for Geriatric Diseases, Department of Geriatrics, Chongqing General Hospital, Chongqing University, Chongqing 401147, China

**Keywords:** *Verruconis gallopava*, immunocompetent, pneumonia, amphotericin B, triazole

## Abstract

*Verruconis* species are thermophilic, darkly pigmented fungi commonly found in hot environments. Despite their environmental ubiquity, fewer than fifty human infections have been reported, with *V. gallopava* responsible for most cases. While infections primarily occur in immunocompromised individuals, only six cases in immunocompetent patients have been documented. We describe a case of pulmonary *Verruconis* infection in a 75-year-old immunocompetent woman. Despite broad-spectrum antifungal treatments, including liposomal amphotericin B and voriconazole, the patient’s condition deteriorated. Bronchoalveolar lavage (BAL) revealed hyphal forms, and fungal culture identified a *Verruconis* species. Antifungal susceptibility tests showed low minimal inhibitory concentrations (MICs) for amphotericin B (1 μg/mL) and voriconazole (0.5 μg/mL). Clinical manifestations of *Verruconis* infection in immunocompetent pneumonia patients are non-specific. Structural lung disease was identified as the primary risk factor in such hosts. BAL fungal cultures and metagenomics are valuable tools in diagnosing rare fungal infections. Treatment regimens vary, with amphotericin B and triazoles being the most commonly used antifungal agents. Currently, there are no standardized guidelines for diagnosis or treatment. Further studies are needed to establish clinical protocols.

## 1. Introduction

Invasive fungal infections cause over 1.5 million deaths annually [1], yet data on thermophilic molds such as *Verruconis* species remain sparse. *V. gallopava* (formerly *Ochroconis gallopava*) is a thermophilic, dematiaceous mold that causes rare infections in humans, mainly affecting immunocompromised individuals. It is commonly found in hot environments [2] and can infect animals, causing encephalitis [3]. Few cases of infection in immunocompetent patients have been reported [4,5,6,7,8,9]. Infections occur via inhalation of fungal spores or contact, typically affecting the lungs. Here, we describe a case of *Verruconis* pulmonary infection and review other immunocompetent cases.

## 2. Case Presentation

**Figure 1:**
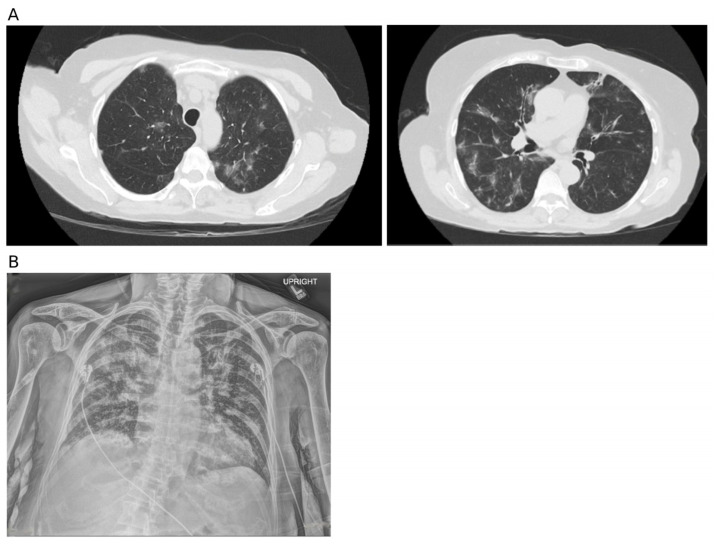
First chest CT scan 7 days before admission, and chest X-ray on the first day of admission. (**A**). The CT scan was performed 7 days before admission. Ground-glass opacities, patchy shadows, and linear shadows were scattered throughout both lungs, with blurred boundaries and partial fusion. Mild bronchial dilatation shadows were seen in some lesions, suggesting infectious lesions in both lungs. (**B**). Chest X-ray on the first day of admission, patchy and nodular high-density blurred shadows distributed diffusely in the lung, the boundaries were unclear and merged with each other; the lungs are similarly expanded to prior with confluent airspace opacity of the right suprahilar space concerning pneumonia.

A 75-year-old woman presented with a week-long history of productive cough, mild shortness of breath, and chest tightness (without fever), which worsened over three days. Eight days prior, her chest computed tomography (CT) scan revealed bilateral scattered light infiltrates, ground-glass opacities, and right middle lobe bronchiectasis (Figure 1A). She visited the emergency department (ED) in our facility on day 0 due to progressing symptoms.

The patient had a past medical history of polymyalgia rheumatica (stable for the past six months, former intermittent steroid use), osteoporosis and Parkinson’s disease, and malnutrition due to medication side effects. She reported pulmonary *Mycobacterium avium complex* (MAC) infection and *Aspergillus* infection 15 years ago, both of which were fully treated.

On physical examination, the patient was tachypneic (24 breaths/min), hypoxic (SpO_2_ 80%), and hypotensive (88/72 mmHg). Diffuse crackles were detected in both lungs. Blood tests in the ED showed a normal white blood cell count (7.2 × 10^3^/mcl, normal range: 3.9–10.7 × 10^3^/mcl) with a normal absolute neutrophil count (5.08 × 10^3^/mcl, normal range: 1.6–10.7 × 10^3^/mcl). The admission chest X-ray revealed confluent airspace opacity of the right suprahilar space concerning pneumonia (Figure 1B).

The patient was empirically started on ceftriaxone 2000 mg daily intravenously (IV) and azithromycin 500 mg daily IV in the ED (treatment timeline see Table 1). The antibiotic regimen was escalated to vancomycin 10 mg/kg daily IV, cefepime 2000 mg every 12 h IV, and azithromycin 500 mg daily IV after admission. Vancomycin was stopped after a negative nasal methicillin-resistant *Staphylococcus aureus* PCR on day 1. Azithromycin was also discontinued after three days.

The patient’s respiratory status deteriorated; she was intubated on day 1. A broad infectious diseases workup, including serum β-D-glucan, serum *Aspergillus* galactomannan, serum *Cryptococcus* antigen, serum and urine *Histoplasma* antigen, serum and urine *Blastomyces* antigen, serum *Histoplasma*, *Blastomyces*, and *Coccidioides* antibodies, was initiated, all of which returned negative results. Gram stains of sputum revealed rare polymorphonuclear cells with rare oropharyngeal flora. However, no significant organism was isolated from bacterial and acid-fast bacilli (AFB) cultures. Bronchoalveolar lavage (BAL) was collected on day 2, which revealed 76% polymorphonuclear cells, 11% lymphocytes, and 7% eosinophils. The *Aspergillus* Galactomannan antigen in BAL was slightly elevated with an index of 0.57 (normal range ≤ 0.49 index). Grocott methenamine silver stain of BAL revealed hyphae forms on day 4, which confirmed invasive fungal infection in this patient. Fungal culture and *Pneumocystis jirovecii* PCR were ordered on the BAL specimen.

Given the patient’s risk factors, clinical presentation, and history of pulmonary *Aspergillus* infection, antifungal treatment was initiated on day 3 with liposomal amphotericin B at 5 mg/kg daily IV. Atovaquone 750 mg twice daily was also prescribed to cover possible *P. jirovecii* pneumonia, although it was discontinued on day 6 following a BAL negative *P. jirovecii* PCR. Her antibiotics were adjusted to vancomycin per pharmacy dosing IV and piperacillin/tazobactam 3.375 g every 8 h IV on day 3 because of clinical deterioration. Vancomycin was stopped on day 8.

**Figure 2:**
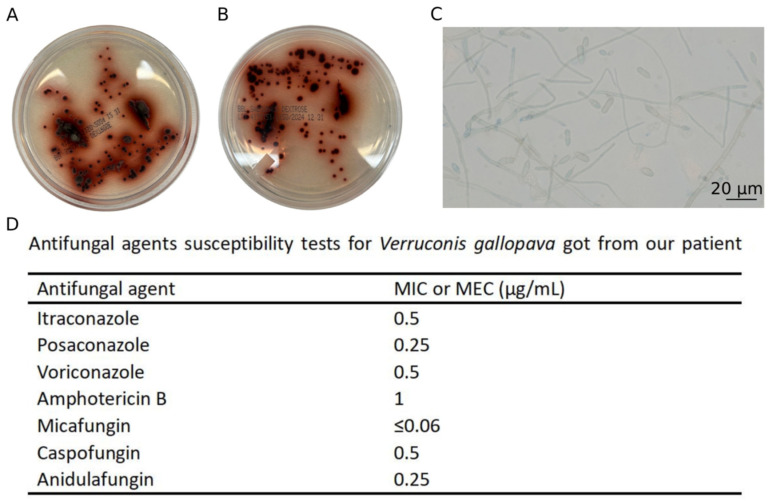
Images of culture plate, staining, and antifungal agent susceptibility test of *Verruconis* species. (**A**,**B**). The maroon pigment of colonies of *Verruconis* species on the front (**A**) and reverse (**B**) sides of the Sabouraud dextrose agar plate. (**C**). Lactophenol cotton blue stain (×400) showed sparse septate hyphae, slender, pointed conidiophores with one or two clavate, two-celled conidia at the tip of the denticles. (**D**). Antifungal susceptibility test results of the *Verruconis* isolate.

On day 6, a mold grew from the BAL fungal culture, which showed tobacco-brown pigment on both the front and back sides of the plate, with dark brown pigment diffused into the Sabouraud dextrose agar (SDA) (Figure 2A,B). Lactophenol blue staining of the mold showed septate, pigmented hyphae, slender, pointed, and pigmented conidiophores with one or two clavate, two-celled conidia at the tip of the denticles (Figure 2C). The isolate demonstrated healthy growth at 42 °C. Based on the macroscopic and microscopic characteristics, as well as its thermophilic feature, the mold was identified as a *Verruconis* species on day 10. The isolate was also sent to ARUP Laboratories for sequencing-based identification and susceptibility testing. Interestingly, DNA sequencing targeting the partial internal transcribed spacer (ITS) region at ARUP failed to definitively identify the organism using a quality-controlled database (because of the absence of a reference sequence of the organism in the database). However, the sequence showed the closest match to a *Verruconis* species when analyzed using NCBI BLAST (https://blast.ncbi.nlm.nih.gov/Blast.cgi (accessed on 22 July 2025)). The results of antifungal susceptibility tests (Figure 2D) suggested relatively low minimal inhibitory concentrations (MICs) with all triazoles tested.

**Figure 3:**
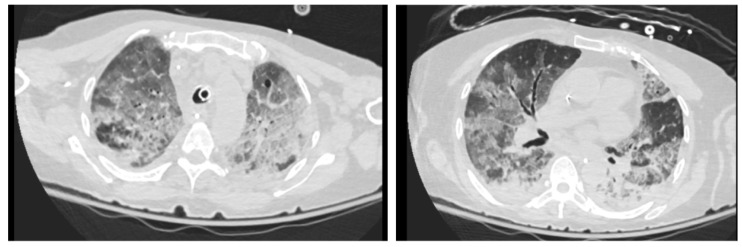
CT scan on the 10th day of admission. CT scan on the 10th day of admission, a “crazy paving” pattern markedly increased in severity. Increase in component of consolidative opacities, predominantly peribronchovascular and regions of ground-glass, air bronchogram seen in the lesion, no discrete cavitation.

Despite active antifungal and antibacterial treatment, the patient’s respiratory status did not improve. A repeated CT scan performed on day 10 suggested progressive diffuse mixed ground-glass opacities with intralobular septal thickening and consolidations (Figure 3). Voriconazole was added for antifungal treatment along with amphotericin B on day 10 (4 mg/kg every 12 h with two loading doses at 6 mg/kg every 12 h orally). Unfortunately, the patient worsened, and she died on day 16.

## 3. Discussion and Conclusions

### 3.1. Literature Review of Immunocompetent Cases

A literature review (1986–2023) identified fewer than 50 reported cases of *Verruconis* infection or synonymous species in humans, with *V. gallopava* responsible for most of the infections [10], including six immunocompetent patients (Supplementary Table S1). Our analysis of the five lung infections (plus our own) revealed key patterns: four had bronchiectasis in images, and one underwent thoracotomy. Common symptoms included productive cough, shortness of breath, and dyspnea on exertion, progressing relatively slowly (except for our patient). Characteristic CT findings included ground-glass infiltrates, multiple opacities/nodules, and bronchiectasis, but notably lacked typical aspergillosis features such as cavities, halo signs, or large nodules, suggesting relatively mild disease progression (Table 2).

### 3.2. Diagnostic Pitfalls in Immunocompetent Hosts

Dematiaceous fungi infections, particularly *Verruconis*, are far less common than *Aspergillus* infections. Key diagnostic challenges emerge when comparing *Verruconis* to *Aspergillus* infections (Table 2). Both fungal infections present atypically in the early stage, complicating timely diagnosis. *Verruconis* infections lack distinct radiological features, such as cavities, halo signs, and large nodules in aspergillosis. In contrast with *Aspergillus* infections, the diagnosis of *Verruconis* lacks established guidelines and specific galactomannan testing.

Current guidelines classify invasive fungal infections in immunocompetent patients as “proven”, “probable”, or “possible” [24,27]. Among reported *Verruconis* cases, three pulmonary and two cutaneous infections were “proven”, while two pulmonary cases were “probable” (Supplementary Table S1). Diagnosis of dematiaceous fungal lung infection presents multiple challenges: tissue biopsies are often contraindicated in critically ill patients, fungal culture methods have suboptimal sensitivity, and radiological imaging features are typically non-specific. As a result, non-culture-based testing methods, such as PCR or metagenomics, are of interest for improving diagnostic sensitivity.

In summary, diagnosing *Verruconis* in immunocompetent hosts is complicated by non-specific symptoms and radiographic features, indolent progression, absence of reliable biomarkers, and lack of established diagnostic criteria. These factors frequently delay diagnosis and treatment initiation. Improved molecular diagnostics and increased clinical awareness are needed to better characterize this emerging fungal pathogen in immunocompetent populations.

### 3.3. MIC-Outcome Paradox of Our Patient: Beyond Laboratory Breakpoints

Despite low MICs for amphotericin B and voriconazole in the *Verruconis* isolates and timely initiation of antifungal therapy, our patient’s condition deteriorated rapidly, resulting in death. Although immunocompetent, the patient had Parkinson’s disease, which caused laryngeal muscle tremors and recurrent microaspiration—a significant risk factor for pulmonary infections.

Notably, the patient had a prior *Mycobacterium avium* complex (MAC) infection, consistent with a previous case report [7]. MAC infection may impair immune function, potentially through altered cytokine release, such as interleukin-10 and tumor necrosis factor-α release, and eicosanoid secretion, such as prostaglandin E2 [28].

We attribute the poor outcome to multiple factors: delayed ED presentation (>1 week after symptom onset), Parkinson-related microaspiration, advanced age, comorbidities, prior steroid use, and a history of fungal infections.

### 3.4. Treatment Options

The broth microdilution method is the reference standard for antifungal susceptibility testing (AST) of molds [29]. Due to limited available data from immunocompetent individuals, susceptibility results from 15 case reports—including this case—involving both immunocompromised and immunocompetent patients were reviewed to better understand treatment options. A total of 15 isolates have been tested, with amphotericin B and triazoles being the most frequently tested antifungal agents (Table 3). The AST results showed low MICs for amphotericin B, echinocandins, and triazoles other than fluconazole. In contrast, high MICs for fluconazole and flucytonsine were observed [8,10,30,31,32,33,34,35,36,37,38,39,40,41]. Additional susceptibility data for *Verruconis* species from laboratory-based AST testing are summarized in Table 4 [3,42,43], showing a trend similar to that seen in the case reports. The novel antifungal agent olorofim has also been tested in vitro and demonstrated a very low MIC.

Combination therapy regimens were frequently used for the treatment of *Verruconis* infections, with amphotericin B combined with one or two triazoles being the most common approach (Table 5). The variety of combination regimens reported from the cases reflects the lack of standardized protocols (Table 5). The survival rate of immunocompetent patients was higher than that of immunocompromised patients (triangles in Table 5, Chi-square test, *p* = 0.0094).

### 3.5. Conclusions

*Verruconis* infections in immunocompetent patients remain rare. Further studies are needed to build standard diagnostic and therapeutic protocols.

## Figures and Tables

**Table 1:** Diagnosis, laboratory tests, and treatment timeline.

Day	Event	Diagnostic Test	Antimicrobial Treatment	
			Start	Stop
−8	Another facility	First CT scan		
0	ED admission	Chest X-ray Broad range virological workup (−) Blood culture (−)	Ceftriaxone 2000 mg qd IV Azithromycin 500 mg qd IV	
0–1			Vancomycin 10 mg/kg qd IV Cefepime 2000 mg q12h IV	Ceftriaxone
1	Admission, intubation	Nasal MRSA PCR (−) Broad range fungal workup (−) Sputum culture (−)		Vancomycin
2	Bronchoscopy	Broad range virological workup (−) *Strongyloides* Ab IgG (−) BAL β-D-glucan (−)		Cefepime Azithromycin
3		Blood culture (−)	Amphotericin B 5 mg/kg qd IV Atovaquone 750 mg bid po Vancomycin pharmacy dosing IV Piperacillin/tazobactam 3.375 g q8h IV	
4		BAL cytology examination revealed hyphae form		
6		BAL fungal culture grew a mold Blood culture (−)		
8				Vancomycin Atovaquone
10		The mold was identified as *Verruconis* species Second CT scan	Voriconazole 4 mg/kg q12h IV	
15		Broad range virological workup (−), blood culture (−)		
16	Death			

CT scan: Computed Tomography scan, ED: Emergency Department, MRSA: Methicillin-Resistant *Staphylococcus Aureus*, BAL: Bronchoalveolar Lavage.

**Table 2:** Comparisons between *Aspergillus* and *Verruconis* infections reported in immunocompetent patients.

	Similarities	Differences		Reference
		*Aspergillus*	*Verruconis*	
Incidence		high	much lower	
Size of conidia		conidia of *Aspergillus fumigatus* are 2–3 μm, probably deposited into the deep lungs	conidia of Verruconis gallopava, around 11–18 × 2.5–4.5 µm, might deposit in the upper airways, especially the oropharyngeal region	Maslov, I.V. [11] Samerpitak, K. [12]
Risk factors	Steroids, chronic structural lung disease, occupational exposure, et al.			Sabir, K. [13]
Clinical presentations	Atypical mild symptoms like cough, dyspnea, fever, fatigue, anorexia, weight loss et al. Some patients have allergic symptoms, like wheezing, shortness of breath	May cause hemoptysis due to angioinvasive feature		Agarwal, R. [14] Patterson, T.F. [15] Ullmann, A.J. [16] Kousha, M. [17] Ledoux, M.P. [18]
Chest CT characteristics	Early signs include: ground-glass infiltration, small nodules	Signs of invasive *Aspergillus* lung infections include: small nodules, typically granulomas formed by inflammatory infiltration; halo sign: a ground-glass halo around a nodule or mass, indicating hemorrhage around the lesion; cavitation or air crescent sign.	Common CT scan findings include ground-glass infiltrates, multiple opacities, multiple pulmonary nodules, and bronchiectasis.	Greene, R. [19] Park, S.Y. [20] Caillot, D. [21] Greene, R.E. [22] Patterson, T.F. [23]
Tests		GM test: BAL > 0.8–1.0 index	GM is not a marker for dematiaceous fungal infection	Bassetti, M. [24]
Diagnosis	Diagnostic classifications: proven; probable; possible; unclassified	Guidelines are available for diagnosis	No specific guideline available	Bassetti, M. [24]
Treatments		First-line treatment for invasive aspergillosis is voriconazole; for chronic pulmonary aspergillosis, itraconazole is the first-line medicine. Monotherapy is usually used.	Given its neurotropic nature, the ability of antifungal agents to penetrate the blood-brain barrier should be considered. Most pulmonary infections in immunocompetent patients have been treated with itraconazole, voriconazole, or a combination regimen of amphotericin B and a triazole.	Patterson, T.F. [15] Sehgal, I.S. [25] Denning, D.W. [26]

CT: Computed Tomography, GM Test: *Aspergillus* Galactomannan Test, BAL: Bronchoalveolar Lavage.

**Table 3:** Summarization of antifungal susceptibility testing results for *Verruconis gallopava* from case reports.

Antifungal Agent	N	Range	MIC50/90	MIC (μg/mL)																						
				0.008	0.015	0.016	0.019	0.023	<0.03	0.03	≤0.06	0.06	<0.12	0.12	<0.125	0.125	0.19	0.25	0.5	<1	1	2	4	16	≥64	References
Amphotericin B	11	0.019–1	0.5/1				1						1					1	3	2	3					Geltner, C [8] Terracol, L. [10] Jennings, Z. [30] Moran, C. [31] El, H.G. [32] Messina, J.A. [33] Meriden, Z. [34] Bernasconi, M. [35] Cardeau-Desangles, I. [36] Mayer, N. [37] Wong, J.S. [38] Bowyer, J.D. [39] Mazur, J.E. [40] Murata, K. [41]
Voriconazole	13	0.008–2	0.5/1	1								1						2	3	1	4	1				
Itraconazole	12	0.016–1	<0.125/0.5			1						2	1	1	1	1		2	2	1						
Posaconazole	12	0.008–1	0.06/0.5	1	1					1		4				2		1	1	1						
Fluconazole	6	16–128	>64/128																						6	
Miconazole	2	1–4	1/4																		1		1			
Isavuconazole	1	N/A	N/A																					1		
Caspofungin	7	<0.03–1	0.5/1						1								1	1	2	1	1					
Anidulafungin	4	0.016–5	0.06/0.5			1						1						1	1							
Micafungin	4	0.023-≤0.06	0.03/≤0.06					1		2	1															
Flucytosine	5	2–64	16/64																		1	1		1	2	
Terbinafine	1	N/A	N/A							1																

Colored numbers indicate the frequency of strains at different concentrations. The yellow color represents the lowest frequency, with the frequency increasing as the color shifts toward deep green.

**Table 4:** Summarization of laboratory-based antifungal susceptibility testing for *Verruconis* species.

Antifungal Agents	Number Isolates Tested	Range	MIC50/90	MIC (μg/mL)														
				0.008	0.016	0.06	0.12	0.125	0.25	0.5	1	2	4	8	>8	64	>64	128
Amphotericin B	10	0.5–2	1/2															
	2	<0.12–0.5	N/A															
	18	0.125–4	0.25/0.5															
Voriconazole	10	0.5–4	2/2															
	2	0.25	N/A															
	18	0.5–2	1/2															
Posaconazole	10	0.125–0.5	0.25/0.25															
	2	0.125–0.5	N/A															
	18	<0.016–4	0.031/0.125															
Itraconazole	2	0.06–0.12	N/A															
	18	0.016–4	0.125/0.5															
Anidulafungin	10	0.06–0.125	0.125/0.25															
	18	0.016–0.125	0.031/0.063															
Fluconazole	2	128	N/A															
	18	4–>64	64/>64															
Isavuconazole	10	4–>8	N/A															
Flucytosine	18	0.5–64	4/32															
Olorofim	10	0.008–0.125	0.015/0.06															
Caspofungin	18	0.25–1	0.5/1															

Colorful stripes indicate the ranges of MICs for different antifungal agents. References: Seyedmousavi, S. [3], Halliday, C.L. [42], Halliday, C.L. [43].

**Table 5:** Mixed antifungal regimen retrieved from case reports.

Antifungal Regimens	Infection Site	Outcome	Reference
Amphotericin B	Lung	Death	El, H.G. [32]
	Lung	Survived	Mancini, M.C. [44]
	Brain	Death	Rossmann, S.N. [45]
Voriconazole	Lung ^Δ^	Survived	Hollingsworth, J.W. [4]
	Lung	Survived	Shoham, S. [46]
	Lung	Survived	Qureshi, Z.A. [47]
	Lung + cutaneous	Survived	Brokalaki, E.I. [48]
	Lung + brain + kidney + muscle	Death	Murata, K. [41]
Itraconazole	Lung ^Δ^	Survived	Odell, J.A. [6]
	Lung ^Δ^	N/A	Bravo, J.L.O. [7]
	Lung	Survived	Bernasconi, M. [35]
	Lung	Survived	Qureshi, Z.A. [47]
	Lung	Survived	Shoham, S. [46]
Flucytosine	Subcutaneous	Death	Fukushiro, R. [49]
Posaconazole	Lung	Survived	Moran, C. [31]
Amphotericin B + Itraconazole	Lung	Survived	Jenney, A. [50]
	Brain	Survived	Qureshi, Z.A. [47]
	Brain	Death	Kralovic, S.M. [51]
	Brain	Death	Qureshi, Z.A. [47]
	Lung	Survived	Zhao, J. [52]
	Cutaneous ^Δ^	N/A	Kumaran, M.S. [5]
	Lung+fungemia	Survived	Qureshi, Z.A. [47]
	Lung+subcutaneous	Survived	Burns, K.E. [53]
Amphotericin B + Voriconazole	Lung	Death	Mayer, N. [37]
	Lung	Survived	Qureshi, Z.A. [47]
	Lung ^Δ^	Death	Our case
	Peritoneum	Survived	Wong, J.S. [38]
	Lung + fungemia + brain	Death	Qureshi, Z.A. [47]
	Lung + brain + cutaneous	Death	Cardeau-Desangles, I. [36]
Voriconazole + Caspofungin	Lung + brain + subcutaneous	Survived	Boggild, A.K. [54]
Voriconazole + Posaconazole	Lung	Survived	Meriden, Z. [34]
	Lung	Survived	Terracol, L. [10]
Itraconazole + Terbinafine	Cutaneous ^Δ^	N/A	Verma, D.G. [9]
Amphotericin B + Voriconazole + Fluconazole	Lung	Survived	Shoham, S. [46]
Amphotericin B + Voriconazole + Itraconazole	Lung ^Δ^	Survived	Geltner, C. [8]
	Lung + brain + spleen	Survived	Wang, T.K. [55]
Amphotericin B + Voriconazole + Posaconazole	Lung + eye	Survived	Kim, E.L. [56]
Amphotericin B + Voriconazole + Micafungin	Lung	Death	Messina, J.A. [33]
Amphotericin B + Itraconazole + Flucytosine	Brain	Survived	Vukmir, R.B. [57]
	Lung + brain + cutaneous	Survived	Mazur, J.E. [40]
	Lung + brain + thyroid	Survived	Malani, P.N. [58]
	Lung + brain + cutaneous	Survived	Moran, C. [31]
Amphotericin B + Itraconazole + Fluconazole	Eye	Death	Bowyer, J.D. [39]
Amphotericin B + Fluconazole + Flucytosine	Brain	Death	Sides, EH Rd [59]
Amphotericin B + Voriconazole + Terbinafine + Anidulafungin	Lung + heart + brain + fungemia + cutaneous	Death	Jennings, Z. [30]
Amphotericin B + Itraconazole + Flucytosine + Terbinafine	Lung + brain + muscle	Death	Fukushima, N. [60]

^Δ^: immunocompetent case.

## Data Availability

The data presented in this study are available on request from the corresponding authors due to the need to protect the privacy of the patient.

## Supplementary Materials

The following supporting information can be downloaded at: https://www.mdpi.com/article/10.3390/jof11090634/s1, Table S1: Characters of immunocompetent patients.

## Abbreviations

CT	Computed tomography
ED	Emergency department
MAC	Mycobacterium avium complex
AFB culture	Acid-fast bacilli
BAL	Bronchoalveolar lavage
MIC	Minimal inhibitory concentration

## References

[1] Denning D.W. (2024). Global incidence and mortality of severe fungal disease. Lancet Infect. Dis..

[2] Giraldo A., Sutton D.A., Samerpitak K., de Hoog G.S., Wiederhold N.P., Guarro J., Gene J. (2014). Occurrence of Ochroconis and Verruconis species in clinical specimens from the United States. J. Clin. Microbiol..

[3] Seyedmousavi S., Samerpitak K., Rijs A.J., Melchers W.J., Mouton J.W., Verweij P.E., de Hoog G.S. (2014). Antifungal susceptibility patterns of opportunistic fungi in the genera Verruconis and Ochroconis. Antimicrob. Agents Chemother..

[4] Hollingsworth J.W., Shofer S., Zaas A. (2007). Successful treatment of *Ochroconis gallopavum* infection in an immunocompetent host. Infection.

[5] Kumaran M.S., Bhagwan S., Savio J., Rudramurthy S.M., Chakrabarti A., Tirumalae R., Abraham A. (2015). Disseminated cutaneous *Ochroconis gallopava* infection in an immunocompetent host: An unusual concurrence—A case report and review of cases reported. Int. J. Dermatol..

[6] Odell J.A., Alvarez S., Cvitkovich D.G., Cortese D.A., Mccomb B.L. (2000). Multiple lung abscesses due to *Ochroconis gallopavum*, a dematiaceous fungus, in a nonimmunocompromised wood pulp worker. Chest.

[7] Bravo J.L.O., Ngauy V. (2004). *Ochroconis gallopavum* and Mycobacterium Avium Intracellulare in an immunocompetent patient. Chest.

[8] Geltner C., Sorschag S., Willinger B., Jaritz T., Saric Z., Lass-Florl C. (2015). Necrotizing mycosis due to *Verruconis gallopava* in an immunocompetent patient. Infection.

[9] Verma D.G. (2018). Primary cutaneous facial phaeohyphomycosis due to *Verruconus gallopava* (*Ochroconus gallopava*) in an immunocompetent woman from the SubHimalayas—A case report and literature review. J. Med. Sci. Clin. Res..

[10] Terracol L., Hamane S., Euzen V., Denis B., Bretagne S., Delliere S. (2024). Phaeohyphomycosis Due to *Verruconis gallopava*: Rare Indolent Pulmonary Infection or Severe Cerebral Fungal Disease?. Mycopathologia.

[11] Maslov I.V., Bogorodskiy A.O., Pavelchenko M.V., Zykov I.O., Troyanova N.I., Borshchevskiy V.I., Shevchenko M.A. (2021). Confocal Laser Scanning Microscopy-Based Quantitative Analysis of Aspergillus fumigatus Conidia Distribution in Whole-Mount Optically Cleared Mouse Lung. J. Vis. Exp..

[12] Samerpitak K., Van der Linde E., Choi H.J., Gerrits Van Den Ende A.H.G., Machouart M., Gueidan C., de Hoog G.S. (2014). Taxonomy of Ochroconis, genus including opportunistic pathogens on humans and animals. Fungal Divers..

[13] Khan S., Bilal H., Shafiq M., Zhang D., Awais M., Chen C., Khan M.N., Wang Q., Cai L., Islam R. (2024). Distribution of Aspergillus species and risk factors for aspergillosis in mainland China: A systematic review. Ther. Adv. Infect. Dis..

[14] Agarwal R., Muthu V., Sehgal I.S., Dhooria S., Prasad K.T., Aggarwal A.N. (2022). Allergic Bronchopulmonary Aspergillosis. Clin. Chest Med..

[15] Patterson T.F., Thompson G.R., Denning D.W., Fishman J.A., Hadley S., Herbrecht R., Kontoyiannis D.P., Marr K.A., Morrison V.A., Nguyen M.H. (2016). Practice Guidelines for the Diagnosis and Management of Aspergillosis: 2016 Update by the Infectious Diseases Society of America. Clin. Infect. Dis..

[16] Ullmann A.J., Aguado J.M., Arikan-Akdagli S., Denning D.W., Groll A.H., Lagrou K., Lass-Florl C., Lewis R.E., Munoz P., Verweij P.E. (2018). Diagnosis and management of Aspergillus diseases: Executive summary of the 2017 ESCMID-ECMM-ERS guideline. Clin. Microbiol. Infect..

[17] Kousha M., Tadi R., Soubani A.O. (2011). Pulmonary aspergillosis: A clinical review. Eur. Respir. Rev..

[18] Ledoux M.P., Herbrecht R. (2023). Invasive Pulmonary Aspergillosis. J. Fungi.

[19] Greene R. (2005). The radiological spectrum of pulmonary aspergillosis. Med. Mycol..

[20] Park S.Y., Lim C., Lee S.O., Choi S.H., Kim Y.S., Woo J.H., Song J.W., Kim M.Y., Chae E.J., Do K.H. (2011). Computed tomography findings in invasive pulmonary aspergillosis in non-neutropenic transplant recipients and neutropenic patients, and their prognostic value. J. Infect..

[21] Caillot D., Casasnovas O., Bernard A., Couaillier J.F., Durand C., Cuisenier B., Solary E., Piard F., Petrella T., Bonnin A. (1997). Improved management of invasive pulmonary aspergillosis in neutropenic patients using early thoracic computed tomographic scan and surgery. J. Clin. Oncol..

[22] Greene R.E., Schlamm H.T., Oestmann J.W., Stark P., Durand C., Lortholary O., Wingard J.R., Herbrecht R., Ribaud P., Patterson T.F. (2007). Imaging findings in acute invasive pulmonary aspergillosis: Clinical significance of the halo sign. Clin. Infect. Dis..

[23] De Pauw B., Walsh T.J., Donnelly J.P., Stevens D.A., Edwards J.E., Calandra T., Pappas P.G., Maertens J., Lortholary O., Kauffman C.A. (2008). Revised definitions of invasive fungal disease from the European Organization for Research and Treatment of Cancer/Invasive Fungal Infections Cooperative Group and the National Institute of Allergy and Infectious Diseases Mycoses Study Group (EORTC/MSG) Consensus Group. Clin. Infect. Dis..

[24] Bassetti M., Azoulay E., Kullberg B.J., Ruhnke M., Shoham S., Vazquez J., Giacobbe D.R., Calandra T. (2021). EORTC/MSGERC Definitions of Invasive Fungal Diseases: Summary of Activities of the Intensive Care Unit Working Group. Clin. Infect. Dis..

[25] Sehgal I.S., Dhooria S., Prasad K.T., Muthu V., Aggarwal A.N., Chakrabarti A., Agarwal R. (2021). Anti-fungal agents in the treatment of chronic pulmonary aspergillosis: Systematic review and a network meta-analysis. Mycoses.

[26] Denning D.W., Cadranel J., Beigelman-Aubry C., Ader F., Chakrabarti A., Blot S., Ullmann A.J., Dimopoulos G., Lange C. (2016). Chronic pulmonary aspergillosis: Rationale and clinical guidelines for diagnosis and management. Eur. Respir. J..

[27] Donnelly J.P., Chen S.C., Kauffman C.A., Steinbach W.J., Baddley J.W., Verweij P.E., Clancy C.J., Wingard J.R., Lockhart S.R., Groll A.H. (2020). Revision and Update of the Consensus Definitions of Invasive Fungal Disease From the European Organization for Research and Treatment of Cancer and the Mycoses Study Group Education and Research Consortium. Clin. Infect. Dis..

[28] Barrow W.W. (1997). Processing of mycobacterial lipids and effects on host responsiveness. Front. Biosci..

[29] Subcommittee on Antifungal Susceptibility Testing (AFST) of the ESCMID European Committee for Antimicrobial Susceptibility Testing (2008). EUCAST Technical Note on the method for the determination of broth dilution minimum inhibitory concentrations of antifungal agents for conidia-forming moulds. Clin. Microbiol. Infect..

[30] Jennings Z., Kable K., Halliday C.L., Nankivell B.J., Kok J., Wong G., Chen S.C. (2017). *Verruconis gallopava* cardiac and endovascular infection with dissemination after renal transplantation: Case report and lessons learned. Med. Mycol. Case Rep..

[31] Moran C., Delafield N.L., Kenny G., Asbury K.L., Larsen B.T., Lambert K.L., Patron R.L. (2019). A case of *Verruconis gallopava* infection in a heart transplant recipient successfully treated with posaconazole. Transpl. Infect. Dis..

[32] El H.G., Palavecino E., Nunez M. (2018). Double invasive fungal infection due to dematiaceous moulds in a renal transplant patient. BMJ Case Rep..

[33] Messina J.A., Wolfe C.R., Hemmersbach-Miller M., Milano C., Todd J.L., Reynolds J., Alexander B.D., Schell W.A., Cuomo C.A., Perfect J.R. (2018). Genomic characterization of recurrent mold infections in thoracic transplant recipients. Transpl. Infect. Dis..

[34] Meriden Z., Marr K.A., Lederman H.M., Illei P.B., Villa K., Riedel S., Carroll K.C., Zhang S.X. (2012). *Ochroconis gallopava* infection in a patient with chronic granulomatous disease: Case report and review of the literature. Med. Mycol..

[35] Bernasconi M., Voinea C., Hauser P.M., Nicod L.P., Lazor R. (2017). *Ochroconis gallopava* bronchitis mimicking haemoptysis in a patient with bronchiectasis. Respir. Med. Case Rep..

[36] Cardeau-Desangles I., Fabre A., Cointault O., Guitard J., Esposito L., Iriart X., Berry A., Valentin A., Cassaing S., Kamar N. (2013). Disseminated *Ochroconis gallopava* infection in a heart transplant patient. Transpl. Infect. Dis..

[37] Mayer N., Bastani B. (2009). A case of pulmonary cavitary lesion due to *Dactylaria constricta* var. gallopava in a renal transplant patient. Nephrology.

[38] Wong J.S., Schousboe M.I., Metcalf S.S., Endre Z.H., Hegarty J.M., Maze M.J., Keith E.R., Seaward L.M., Podmore R.G. (2010). *Ochroconis gallopava* peritonitis in a cardiac transplant patient on continuous ambulatory peritoneal dialysis. Transpl. Infect. Dis..

[39] Bowyer J.D., Johnson E.M., Horn E.H., Gregson R.M. (2000). *Oochroconis gallopava* endophthalmitis in fludarabine treated chronic lymphocytic leukaemia. Br. J. Ophthalmol..

[40] Mazur J.E., Judson M.A. (2001). A case report of a dactylaria fungal infection in a lung transplant patient. Chest.

[41] Murata K., Ogawa Y., Kusama K., Yasuhara Y. (2022). Disseminated *Verruconis gallopava* infection in a patient with systemic lupus erythematosus in Japan: A case report, literature review, and autopsy case. Med. Mycol. Case Rep..

[42] Halliday C.L., Tay E., Green W., Law D., Lopez R., Faris S., Meehan L., Harvey E., Birch M., Chen S. (2024). In vitro activity of olorofim against 507 filamentous fungi including antifungal drug-resistant strains at a tertiary laboratory in Australia: 2020–2023. J. Antimicrob. Chemother..

[43] Halliday C.L., Chen S.C., Kidd S.E., van Hal S., Chapman B., Heath C.H., Lee A., Kennedy K.J., Daveson K., Sorrell T.C. (2016). Antifungal susceptibilities of non-Aspergillus filamentous fungi causing invasive infection in Australia: Support for current antifungal guideline recommendations. Int. J. Antimicrob. Agents.

[44] Mancini M.C., Mcginnis M.R. (1992). Dactylaria infection of a human being: Pulmonary disease in a heart transplant recipient. J. Heart Lung Transplant..

[45] Rossmann S.N., Cernoch P.L., Davis J.R. (1996). Dematiaceous fungi are an increasing cause of human disease. Clin. Infect. Dis..

[46] Shoham S., Pic-Aluas L., Taylor J., Cortez K., Rinaldi M.G., Shea Y., Walsh T.J. (2008). Transplant-associated *Ochroconis gallopava* infections. Transpl. Infect. Dis..

[47] Qureshi Z.A., Kwak E.J., Nguyen M.H., Silveira F.P. (2012). *Ochroconis gallopava*: A dematiaceous mold causing infections in transplant recipients. Clin. Transplant..

[48] Brokalaki E.I., Sommerwerck U., von Heinegg E.H., Hillen U. (2012). *Ochroconis gallopavum* infection in a lung transplant recipient: Report of a case. Transplant. Proc..

[49] Fukushiro R., Udagawa S., Kawashima Y., Kawamura Y. (1986). Subcutaneous abscesses caused by *Ochroconis gallopavum*. J. Med. Vet. Mycol..

[50] Jenney A., Maslen M., Bergin P., Tang S.K., Esmore D., Fuller A. (1998). Pulmonary infection due to *Ochroconis gallopavum* treated successfully after orthotopic heart transplantation. Clin. Infect. Dis..

[51] Kralovic S.M., Rhodes J.C. (1995). Phaeohyphomycosis caused by Dactylaria (human dactylariosis): Report of a case with review of the literature. J. Infect..

[52] Zhao J., Wang Z., Li R., Wang D., Bai Y. (2002). Pemphigus patient with pulmonary fungal infection caused by *Ochroconis gallopava*: The first case report in China. Zhonghua Yi Xue Za Zhi.

[53] Burns K.E., Ohori N.P., Iacono A.T. (2000). *Dactylaria gallopava* infection presenting as a pulmonary nodule in a single-lung transplant recipient. J. Heart Lung Transplant..

[54] Boggild A.K., Poutanen S.M., Mohan S., Ostrowski M.A. (2006). Disseminated phaeohyphomycosis due to *Ochroconis gallopavum* in the setting of advanced HIV infection. Med. Mycol..

[55] Wang T.K., Chiu W., Chim S., Chan T.M., Wong S.S., Ho P.L. (2003). Disseminated ochroconis gallopavum infection in a renal transplant recipient: The first reported case and a review of the literature. Clin. Nephrol..

[56] Kim E.L., Patel S.R., George M.S., Ameri H. (2018). *Ochroconis gallopava* Endophthalmitis Successfully Treated with Intravitreal Voriconazole and Amphotericin B. Retin. Cases Brief Rep..

[57] Vukmir R.B., Kusne S., Linden P., Pasculle W., Fothergill A.W., Sheaffer J., Nieto J., Segal R., Merhav H., Martinez A.J. (1994). Successful therapy for cerebral phaeohyphomycosis due to *Dactylaria gallopava* in a liver transplant recipient. Clin. Infect. Dis..

[58] Malani P.N., Bleicher J.J., Kauffman C.A., Davenport D.S. (2001). Disseminated *Dactylaria constricta* infection in a renal transplant recipient. Transpl. Infect. Dis..

[59] Sides E.R., Benson J.D., Padhye A.A. (1991). Phaeohyphomycotic brain abscess due to *Ochroconis gallopavum* in a patient with malignant lymphoma of a large cell type. J. Med. Vet. Mycol..

[60] Fukushima N., Mannen K., Okamoto S., Shinogi T., Nishimoto K., Sueoka E. (2005). Disseminated *Ochroconis gallopavum* infection in a chronic lymphocytic leukemia: A case report and review of the literature on hematological malignancies. Intern. Med..

